# Seasonal dynamics in a cavity-nesting bee-wasp community: Shifts in composition, functional diversity and host-parasitoid network structure

**DOI:** 10.1371/journal.pone.0205854

**Published:** 2018-10-16

**Authors:** Sergio Osorio-Canadas, Xavier Arnan, Emili Bassols, Narcís Vicens, Jordi Bosch

**Affiliations:** 1 CREAF, Cerdanyola del Vallès, Barcelona, Spain; 2 Parc Natural de la Zona Volcànica de la Garrotxa, Olot, Spain; 3 Servei de Medi Ambient de la Diputació de Girona, Pujada Sant Martí 4–5, Girona, Spain; University of New England, AUSTRALIA

## Abstract

Ecological communities are composed of species that interact with each other forming complex interaction networks. Although interaction networks have been usually treated as static entities, interactions show high levels of temporal variation, mainly due to temporal species turnover. Changes in taxonomic composition are likely to bring about changes in functional trait composition. Because functional traits influence the likelihood that two species interact, temporal changes in functional composition and structure may ultimately affect interaction network structure. Here, we study the seasonality (spring vs. summer) in a community of cavity-nesting solitary bees and wasps (‘hosts’) and their nest associates (‘parasitoids’). We analyze seasonal changes in taxonomic compostion and structure, as well as in functional traits, of the host and parasitoid communities. We also analyze whether these changes result in changes in percent parasitism and interaction network structure. Our host and parasitoid communities are strongly seasonal. Host species richness increases from spring to summer. This results in important seasonal changes in functional composition of the host community. The spring community (almost exclusively composed of bees) is characterized by large, univoltine, adult-wintering host species. The summer community (composed of both bees and wasps) is dominated by smaller, bivoltine, prepupa-wintering species. Host functional diversity is higher in summer than in spring. Importantly, these functional changes are not only explained by the addition of wasp species in summer. Functional changes in the parasitoid community are much less pronounced, probably due to the lower parasitoid species turnover. Despite these important taxonomic and functional changes, levels of parasitism did not change across seasons. Two network metrics (generality and interaction evenness) increased from spring to summer. These changes can be explained by the seasonal increase in species richness (and therefore network size). The seasonal shift from a bee-dominated community in spring to a wasp-dominated community in summer suggests a change in ecosystem function, with emphasis on pollination in spring to emphasis on predation in summer.

## Introduction

Biological communities are composed of species that interact among themselves in various ways forming complex interaction networks. Ultimately, the structure of these networks reflects ecosystem functioning and community stability [1, 2]. Although we often implicitly treat interaction networks as static entities (“the food web of a given locality”, “the pollination network of a given habitat”), interactions show high levels of variation at various temporal scales. Abiotic conditions such as temperature and precipitation fluctuate periodically in more or less predictably ways throughout the year (seasonality), and the timing of the life cycle of organisms (phenology) has evolved in response to these changes [3–5]. Consequently, seasonality often results in important changes in community composition [6–8]. Changes in community composition are likely to affect interaction composition, but the extent to which changes in community structure translate into changes in network structure is still unclear. Some studies show concomitant changes in community and network structure [9–14], while others have found changes in community structure but little or no changes in network structure [15–16].

Temporal changes in community structure and composition are likely to lead to changes in community functional trait composition [17–19]. Compared to classical taxonomic-based approaches, trait-based approaches provide an improved mechanistic understanding of species–environment relationships [20, 21]. A functional approach is especially important for the study of interaction networks, because functional traits affect species interactions at two levels. First, trait-mediated environmental filtering influences species distribution and abundance, and therefore affects the probability that two species may co-occur and potentially interact. Second, trait-mediated morphological and phenological matching drives interactions between potential partners [22–24], and particular functional traits are related to specialization [25–27]. In aphid-parasitoid networks, food and habitat specialization, mobility, body size, and colony organization are associated with parasitoid specialization [28]. However, it is still unclear how changes in functional trait composition relate to variation in overall network structure.

In this study we analyze the seasonality (spring vs summer) of a community of cavity-nesting solitary bees and wasps (henceforth ‘hosts’) and their nest associates (including parasitoids, cleptoparasites and predators/scavengers; henceforth ‘parasitoids’). Cavity-nesting bees and wasps have been used to study host-parasitoid interactions in different habitats [9, 10, 12, 29] and along various types of gradients [13, 16, 30]. However, the seasonal dynamics of these communities remain largely unexplored. We sampled our community at regular time intervals, which affords us the opportunity to incorporate a temporal dimension to the study of bee-wasp communities and their interactions with parasitoids. We analyze seasonal changes in taxonomic and functional structure and composition of the host-parasitoid community and in the resulting interaction network. Our study area (NE Iberian Peninsula) shows a marked seasonality in climatic conditions, with cool wet springs and hot dry summers (see ‘Material & Methods‘). It also shows seasonality in the availability of food resources for cavity-nesting bees (pollen and nectar) and wasps (insect and spider prey). Pollen and nectar are much more abundant in spring [31, 32], whereas arthropod preys (aphids, caterpillars and spiders) are more abundant in summer [33–36]. For these reasons, and given that solitary bees and wasps usually have short activity periods [37–39], we expect high species and functional trait turnover across seasons. We have three objectives: 1) To analyze seasonal changes in species richness, abundance and composition of the host and parasitoid communities; 2) To establish whether these changes result in changes in community functional structure; 3) To establish whether these changes affect interaction patterns (parasitism rates and network structure).

## Materials and methods

### Ethics statement

Field work was conducted with permission of Parc Natural de la Zona Volcànica de la Garrotxa, and of the various owners of private land in which the study was conducted. Our study does not involve any endangered or protected species.

### Study area and sites

The study area covers a surface of about 100 km^2^ around the city of Olot (Catalonia, NE Spain, 42°11'N, 2°29'E; 443 m above sea level). The climate is Mediterranean with continental influence, and mean annual temperature and cumulative annual precipitation of 13°C and 1000 L/m^2^, respectively. The natural vegetation is a mixed forest with Mediterranean species (*Quercus ilex*) alongside mid-elevation continental species (*Quercus robur*, *Fagus sylvatica*). Urban development and agricultural areas (mainly cereals) are intermixed within the forest matrix forming a complex small-scale mosaic. We selected 14 sites (farms in clearings within the forest matrix). Distance between sites ranged from 1.4 to 13 km.

### Trap-nesting

Cavity-nesting bees and wasps (henceforth CNBW) build nests in pre-established cavities such as abandoned beetle burrows in dead trees, hollow stems, abandoned bee/wasp nests, and holes in rocks. They also use artificial cavities such as cracks and holes in the walls of old human-made buildings. At each site, we placed a trap-nesting station consisting of 7 drilled wood blocks with inserted paper tubes. Each wood block accommodated 25 tubes of a given diameter (2, 3, 4, 5, 6, 7 or 8 mm). Paper tube length was 5 cm for the 2 and 3 mm diameters and 15 cm for the rest. Nesting stations were attached to farm buildings approximately at 1.5 m above the ground, facing SE.

To obtain data on host and parasitoid seasonality, nests were sampled throughout the entire bee-wasp season (early April to late September) in 1991. During this period, we made biweekly visits to each site and replaced filled paper tubes with empty ones to make sure there would be cavities of all diameters available at all times. Tubes containing nests were taken to the laboratory and kept at room temperature 20–25°C until end October, when they were transferred to an unheated storage unit (2–10°C) for wintering. In the following spring, tubes were again exposed to room temperature (22–25°C) to stimulate development. Then, tubes were dissected and their contents (number and identity of hosts and parasitoids) recorded.

### Seasonality

We divided the nesting period into two seasons of equal duration and clearly contrasted meteorological regimes: spring (nests built from early April to late June; mean temperature, 15.2°C; cumulative precipitation, 247 L/m^2^), and summer (early July to late September, 20.8°C, 202 L/m^2^).

### Taxonomic community structure

To describe taxonomic community structure, we used host species richness, host abundance (number of host cells produced), parasitoid species richness and parasitoid abundance (number of parasitized cells) of each site and season.

### Functional community structure and composition

For each host and parasitoid species we compiled information on five functional traits. Hosts were characterized based on: 1) body size, 2) larval diet, 3) wintering stage, 4) voltinism, and 5) nest-building material (see description and methodology in S1 and S2 Tables). Parasitoids were characterized based on: 1) body size, 2) parasitic behavior, 3) wintering stage, 4) voltinism, and 5) gregariousness (see description and methodology in S1 and S3 Tables). These traits are generally assumed to be important for species performance [40, 41] and are likely to affect the establishment of interactions with other species (S1 Table).

We characterized the functional composition of the host and parasitoid communities at each site and season by computing two functional indices: 1) Trait average; indicative of the most common trait in a community. For continuous traits (body size), trait average was computed as the weighted community mean (mean of the trait values of all species in the community weighted by their abundance). In the case of categorical traits, each level of the trait was converted into a separate variable and the proportion of individuals of each species accounting for each level was computed [42, 43]; and 2) Functional dispersion (FDis); which provides a measure of functional trait diversity and reflects the extent to which species within a community differ in their traits [42]. FDis was computed for each single trait and for all traits together. This index quantifies the mean distance of each species from its community average (or centroid in a multivariate space when considering several traits together). FDis is mathematically independent of species richness and was calculated as an abundance-weighted (quantitative) metric [44, 45]. To calculate trait average and FDis indices, we used the function ‘dbFD’ in ‘FD’ package [42, 43] for R version 3.3.1 [46]. We used “lingoes” correction for non-Euclidean distances [42, 47].

### Percent parasitism and host-parasitoid network structure

Percent parasitism is expressed as the percentage of cells that were parasitized. To describe host-parasitoid network structure, we first built a spring and a summer host-parasitoid network for each of the 14 sites (28 networks). We then computed the following quantitative metrics related to network specialization: 1) generality (weighted average number of host species per parasitoid) [48]; 2) vulnerability (weighted average number of parasitoid species per host) [48]; 3) interaction evenness (Shannon’s diversity of interactions / ln (hosts richness * parasitoid richness) [49]; and 4) the specialization index H_2_', a measure of the degree of complementary specialization at the network level. This metric, which accounts for the interaction frequency (number of parasitized brood cells) of each species, is not affected by network size and ranges between 0 (maximum generalization) and 1 (maximum specialization) [50]. Three of our 28 networks were too small to obtain a reliable computation of network metrics and were excluded from all network analyses. Therefore, these analyses were conducted with 11 spring and 14 summer networks. All metrics were calculated with ‘bipartite’ v.1.16 [49] for R.

### Statistical analyses

#### Effects of seasonality on host and parasitoid taxonomic community structure and composition

We used general linear mixed models (General LMM) (*lme* function, ‘nlme’ package [51] for R) to analyze the effects of season on each response variable (host abundance, host richness, parasitoid abundance, parasitoid richness). To account for repeated temporal measures (spring and summer), we included site as a random factor. Some studies have found parasitoid richness and parasitoid abundance to be correlated to host richness and/or abundance [10, 29, 52, 53]. Thus, analyses of parasitoid-related variables were repeated controlling for potential host effects. Host abundance was not affected by season (see results). Therefore, we only used host richness as a controlling variable. Host richness was strongly related to season (see results). Thus, it could not be included as a covariate with season in the same model. Therefore, to extract the effects of host richness on parasitoid richness and abundance we first built a General LMM with the response variable (parasitoid richness or parasitoid abundance, respectively) and only host richness as the explanatory variable, and then we extracted the residuals of these models as new response variables.

To explore the effects of season on taxonomic community composition, we used permutational multivariate analysis of variance (PERMANOVA) (*adonis* function, ‘Vegan’ package [54] for R), separately for hosts and parasitoids. Abundance data for each site in both seasons were square-root transformed, and distance matrices were calculated with the Bray-Curtis dissimilarity index. We run 9999 permutations per test. Because we used the same sites along the two seasons, data were grouped by site using the function strata, which constrains the number of permutations within groups (sites) similarly to a random factor. We also ran qualitative (presence/absence) versions of these PERMANOVAs to evaluate if possible seasonal community shifts are just due to changes in relative abundances, or mainly due to species turnover. To visualize differences in community composition among sites in each season (separately for hosts and parasitoids) we conducted multidimensional scaling (NMDS) based on the Bray-Curtis dissimilarity index (*metaMDS* function, ‘Vegan’ package for R). We selected the number of dimensions (k) considering ‘stress’ values (a measure of goodness of fit based on the Bray-Curtis dissimilarity index and distance in graphical representation).

#### Effects of seasonality on community functional composition

We used General LMMs to analyze the effects of season (spring vs summer) on each functional response variable: 1) trait average index for each trait (for categorical traits that only had two levels, analyses were conducted just for one level); and 2) FDis for each trait and for the entire set of traits. All analyses were conducted separately for hosts and parasitoids.

#### Effects of seasonality on parasitism and network structure

We again used General LMMs to analyze the effect of season (spring vs summer) on percentage of parasitism and on network metrics (vulnerability, generality, interaction evenness and H_2_'). As with taxonomic community structure variables, percent parasitism and network metrics may be affected by certain covariates (host richness, host abundance, parasitoid richness; [10, 29, 52, 53, 55]). Thus, we repeated these analyses controlling for potential effects of these covariates. Host abundance was not related to season in our study (see results), and parasitoid richness was related to season through the effect of host richness (see results). Therefore, we used host richness as a controlling variable. However, host richness was strongly related to season (see results), and thus, could not be included as a covariate with season in the same model. To extract the effects of host richness on percent parasitism, we first built a General LMM with the response variable (percent parasitism) and only host richness as the explanatory variable, and then we used the residuals of this model in a new analysis with season as the response variable. Vulnerability, generality and interaction evenness may be affected by network size [9, 29, 49, 56]. For this reason, we tried to use network size (host richness + parasitoid richness) as a controlling variable in the seasonality analyses. However, network size and season were related (mean network size ± SE: spring, 9.3 ± 1.2; summer, 15.0 ± 0.8; General LMM: F_1,10_ = 26.8, *P* = 0.0004), and therefore could not be used in the same analysis. As a result, we followed the same procedure as for percent parasitism: we first built a General LMM for each of the response variable (vulnerability, generality, interaction evenness) with network size as explanatory variable, and then we extracted the residuals of these three models as new response variables in three new analyses with season as a response variable. In all General LMMs, adjusted-pseudo R^2^ was calculated based on Likelihood-ratio tests with the *r*.*squaredLR* function (‘MuMIn’ package, [57] for R).

In all analyses we checked the residuals for the assumptions of normality and homoscedasticity. Transformations were applied as needed (see S5 Table).

## Results

### General description of the community

We obtained 1491 nests amounting to 5703 cells. About half of the nests (57.9%) corresponded to 16 bee species (13 Megachilidae, 3 Colletidae) (S4 Table). The remaining nests corresponded to 11 wasp species (6 Crabronidae, 4 Vespidae, 1 Sphecidae) (S4 Table). We also found 19 species of parasitoids (S4 Table). The number of nests obtained was similar in the two seasons (719 in spring, 772 in summer), but we obtained more cells in spring (3485) than in summer (2218).

### Effects of seasonality on community taxonomic structure and composition

**Fig 1:**
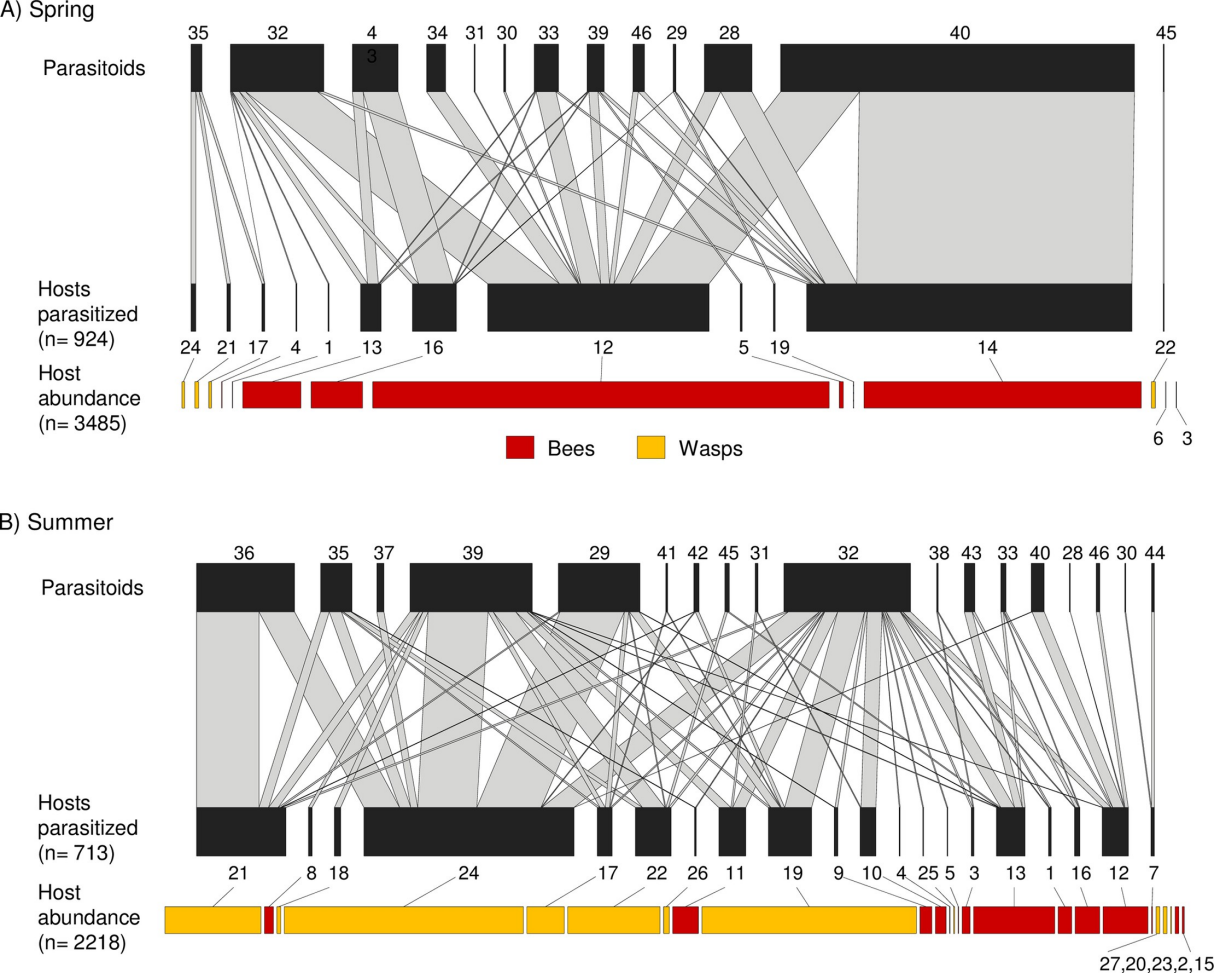
Effect of season on host-parasitoid networks. Spring (A) and summer (B) host-parasitoid network (data from 14 sites lumped together). Numbers correspond to species names in S4 Table. Width of grey bands denotes interaction strength (number of cells parasitized). Width of red and yellow bars indicates host abundance (number of cells produced). Note different scales (number of cells) in spring and summer networks.

There were no significant differences between seasons in host abundance, but host richness was significantly higher in summer (mean ± SE: 8.4 ± 0.5) than in spring (3.9 ± 0.6) (Table 1, Fig 1). Wasps were very rare in spring. Therefore, the increase in host richness in summer might be simply due to the addition of wasp species. To test this possibility, we repeated the richness analyses only with bee hosts, and there were no seasonal differences in mean bee richness (spring: 3.1 ± 0.5, summer: 3.7 ± 0.3; General LMM: reference level: summer; *t* = 1.26, *P* = 0.23). Therefore, the increase in host richness was due to the addition of wasp species in summer. We found no differences in parasitoid abundance between seasons (Table 1). Parasitoid richness tended to be higher in summer (6.6 ± 0.5) than in spring (5.4 ± 0.7), but once controlled for host richness, the effect of season on parasitoid richness was clearly non-significant (Table 1).

**Table 1:** Effect of season on various community and network metrics.

Response variable	*t*	Pseudo-R^2^	*P*	Controlled variable	*t*	Pseudo-R^2^	*P*
Host abundance	-0.78	0.02	0.45	None	-	-	-
Host richness	11.4	0.50	**<0.0001**	None	-	-	-
Parasitoid abundance	-0.27	0.003	0.79	Host richness	1.20	0.05	0.25
Parasitoid richness	1.93	0.12	0.076	Host richness	1.51	0.08	0.16
Percent parasitism	0.067	0.001	0.95	Host richness	0.82	0.03	0.43
Vulnerability	0.34	0.01	0.74	Network size	0.97	0.04	0.35
Generality	3.56	0.32	**0.005**	Network size	1.62	0.10	0.13
Interaction evenness	2.27	0.18	**0.046**	Network size	1.40	0.08	0.19
H_2_’	-0.65	0.07	0.53	None	-	-	-

Summary of the General linear mixed model outputs analyzing the effect of season (reference level: summer) on various community and network metrics. Six of the analyses (parasitoid abundance, parasitoid richness, percentage of parasitism, vulnerability, generality and interaction evenness) were repeated controlling for the effects of certain covariates (controlled variable). Significant values are marked in bold.

**Fig 2:**
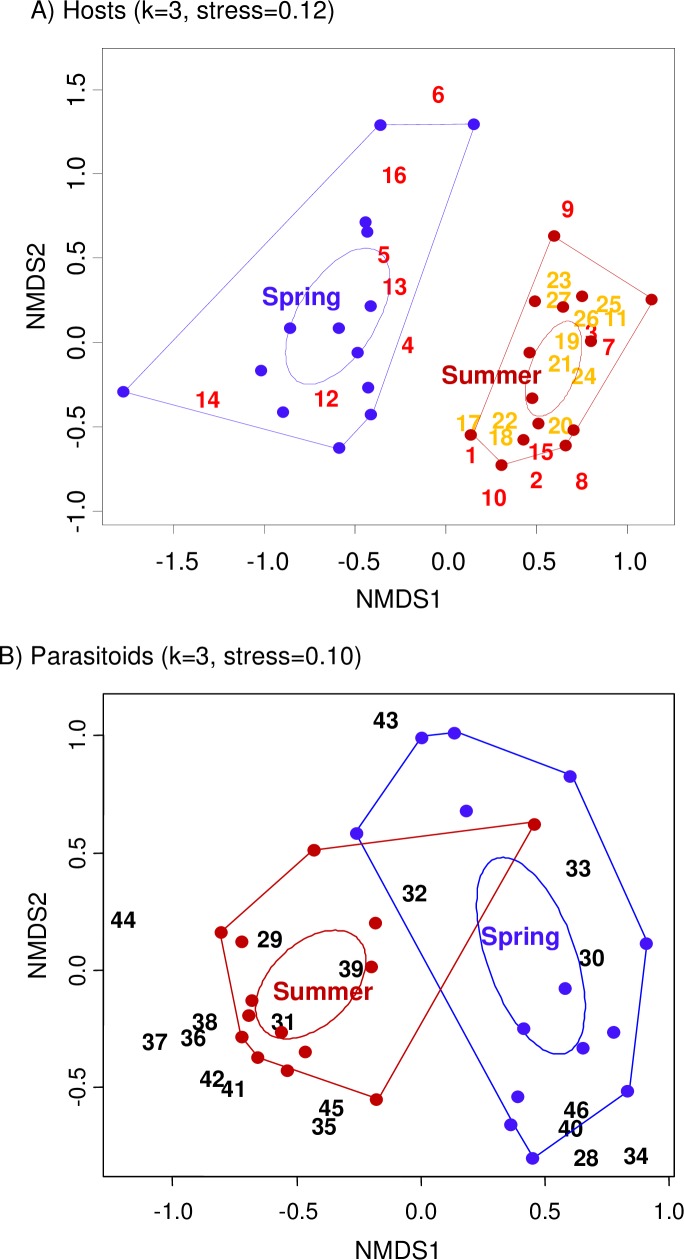
Effect of season on community composition. Nonmetric multidimensional scaling (NMDS) of (A) host and (B) parasitoid community composition in each season and site. Dots represent seasons (blue: spring; red: summer). Numbers represent species codes (S4 Table; red: bees; orange: wasps; black: parasitoids). Polygons encompass all sites within a season. Ellipses represent 0.95% confidence intervals. Only two of the three dimensions obtained in the analyses (k = 3) are displayed.

Bee activity began in early spring and continued through the summer. Bee composition showed a strong seasonality, with two species occurring only in spring, seven in spring and summer, and seven only in summer. Wasp activity did not start until late spring. Even then, the wasp community also showed a strong seasonality. Five wasp species occurred in late spring and summer, and six only in summer. Consequently, differences between seasons in host community composition were highly significant (Table 2A, Fig 2A). These differences were not only due to changes in relative frequencies of host species, but also to species turnover, as PERMANOVA for qualitative (presence/absence) data also yield highly significant differences (Table 2B). Since wasps were very rare in spring, we repeated these analyses only with bee host species, and again we found highly significant differences in community composition between seasons, both with quantitative (Table 2A) and qualitative data (Table 2B).

**Table 2:** Effect of season on community composition.

**A. Quantitative data**						
**Response variable**	**Explanatory variable**	**df**	**Sums of squares**	**F**	**Pseudo-R**^**2**^	***P***
Hosts	Season	1	2.38	8.44	0.24	**0.0001**
Hosts (only bees)	Season	1	1.43	4.28	0.14	**0.0013**
Parasitoids	Season	1	1.98	9.05	0.26	**0.0001**
**B. Qualitative (presence/absence) data**						
**Response variable**	**Explanatory variable**	**df**	**Sums of squares**	**F**	**Pseudo-R**^**2**^	***P***
Hosts	Season	1	1.72	10.40	0.29	**0.0002**
Host (only bees)	Season	1	0.87	4.40	0.14	**0.0003**
Parasitoids	Season	1	1.11	8.49	0.24	**0.0003**

Results of PERMANOVA analyses of community composition using quantitative (A) and qualitative (B) data. Significant values are marked in bold.

The parasitoid community also changed significantly from spring to summer considering both quantitative (Table 2A, Fig 2B) and qualitative data (Table 2B). One parasitoid species was only found in spring, twelve in both spring and summer, and six only in summer. In spring, the parasitoid community was dominated by species exclusively attacking bees (*Cacoxenus indagator* and *Monodontomerus obsoletus;* codes 34 and 40, respectively in Fig 1). In summer, the parasitoid community was dominated by species that either only attacked wasp hosts (*Pyemotes ventricosus*, Sarcophagidae sp. 1 and Sarcophagidae sp. 2; codes 29, 35 and 36, respectively in Fig 1) or attacking both kinds of hosts but showing a clear preference for wasps (*Trichodes alvearius* and *Melittobia acasta;* codes 32 and 39, repectively in Fig 1).

### Effects of seasonality on community functional structure and composition

**Fig 3:**
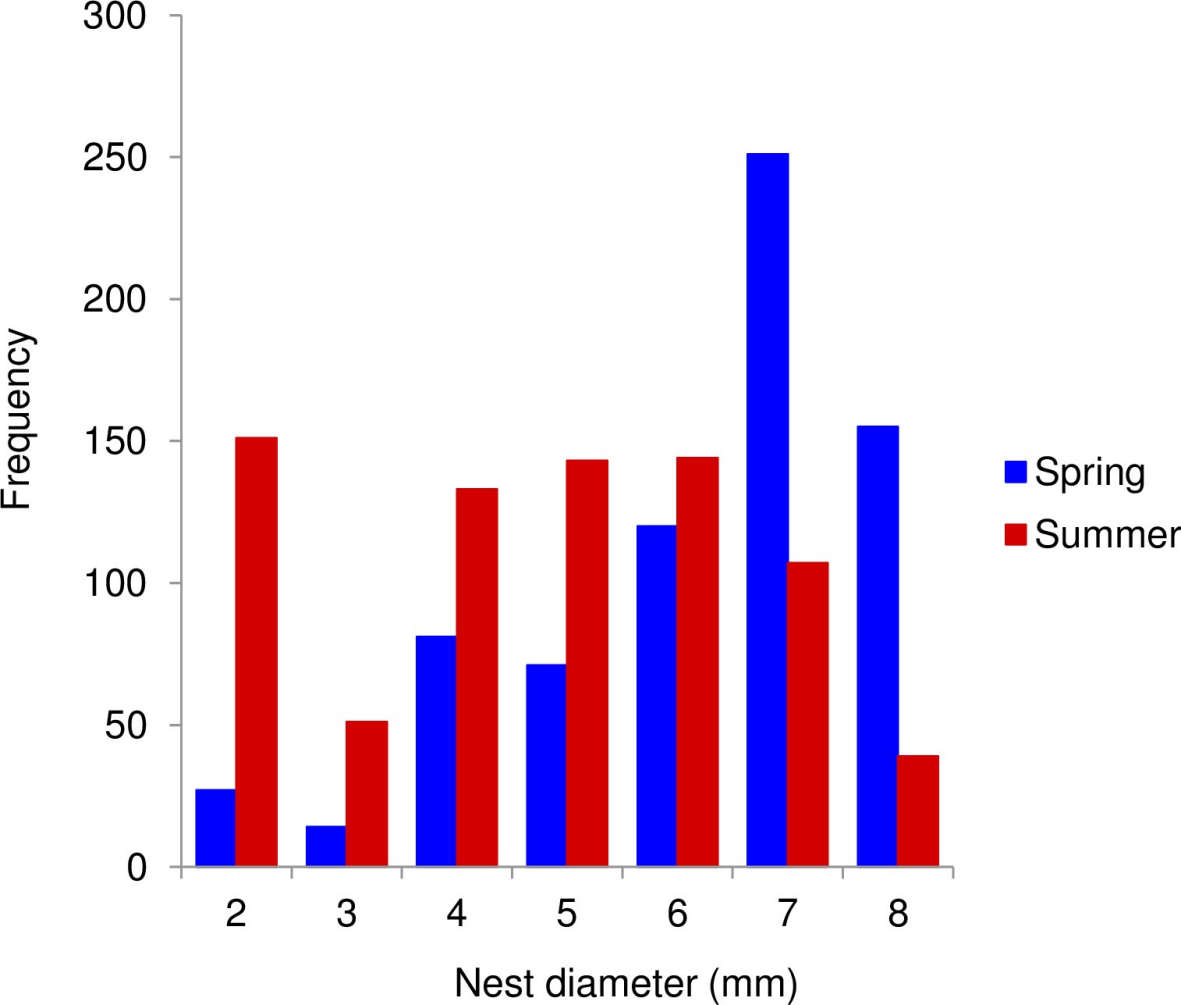
Effect of season on host nest diameter. Distribution of cavity diameters used by hosts for nest construction in spring (n = 719 nests) and summer (n = 768 nests). (X-squared = 272.45, df = 6, P < 0.0001).

Most host traits showed significant shifts from spring to summer (Table 3A). Overall, the spring community was characterized by larger, univoltine, adult-wintering host species, with a pollenivorous larval diet (bees). In contrast, the summer community was dominated by smaller, bivoltine, prepupa-wintering species, mostly with carnivorous larvae (wasps). The decrease in body size from spring to summer resulted in a decrease in the diameter of the cavities used for nesting (spring: 6.25 ± 0.1 mm; summer: 4.72 ± 0.1) (Fig 3). The summer community also had a higher incidence of species building their nests with glandular secretions. The use of other nesting materials did not vary between seasons. Again, because wasps were very rare in spring, we repeated these analyses only for bee species. We obtained similar results for all traits, except for the proportion of species using mud as nesting material, which significantly decreased from spring to summer (Table 3B). Overall, host FDis was higher in summer than in spring, and this pattern did not change when only bees were considered, even though the traits involved were not entirely coincidental (Table 3A and 3B). The parasitoid community showed a different pattern. None of the parasitoid traits considered showed seasonal variation (Table 3C). However, similarly to hosts, overall parasitoid FDis was much higher in summer than in spring (Table 3C).

**Table 3:** Effect of season on trait average and functional dispersion.

**A. Hosts (bees + wasps)**					
		**Trait average**		**FDis**	
**Trait**	**Variable**	***t***	***P***	***t***	***P***
Larval diet	% pollenivorous	-12.9	<0.0001	7.0	**<0.0001**
Body size	Inter-tegular span	-6.6	<0.0001	1.7	0.1
Wintering stage	% prepupa	20.3	<0.0001	3.4	**0.007**
Voltinism	% univoltine	-7.9	<0.0001	1.2	0.3
Nest-building material	% mud	-1.0	0.3	4.4	**0.001**
	% plant material	0.2	0.9		
	% secretions	3.5	0.004		
All traits	-	-	-	4.9	**0.0006**
**B. Hosts (only bees)**					
		**Trait average**		**FDis**	
**Trait**	**Variable**	***t***	***P***	***t***	***P***
Body size	Inter-tegular span	-4.6	0.0005	0.1	0.9
Wintering stage	% prepupa	5.2	0.0002	3.9	**0.004**
Voltinism	% univoltine	-3.0	0.01	2.9	**0.02**
Nest-building material	% mud	-3.1	0.009	1.5	0.2
	% plant material	1.6	0.13		
	% secretions	2.6	0.02		
All traits	-	-	-	3.6	**0.005**
**C. Parasitoids**					
		**Trait average**		**FDis**	
**Trait**	**Variable**	***t***	***P***	***t***	***P***
Parasitic behavior	% cleptoparasite	0.3	0.8	2.0	0.07
	% parasitoid	-0.3	0.8		
	% scavenger	-0.02	0.9		
Body size length	Length	-1.6	0.1	4.4	**0.0009**
Wintering stage	% immature	-0.1	0.9	1.9	0.09
Voltinism	% univoltine	-0.2	0.9	3.0	**0.006**
Gregariousness	% solitary	-1.4	0.2	2.1	0.06
All traits	-	-	-	3.5	**0.005**

Summary of linear mixed model outputs analyzing the effect of season (reference level: summer) on trait average and functional dispersion (FDis, for single traits and for all traits together) in (A) host, (B) bee host and (C) parasitoid communities. Significant values are marked in bold.

### Effects of seasonality on parasitism and network structure

Despite the above-mentioned differences between seasons in taxonomic and functional community structure and composition, there were no significant differences in percent parasitism between spring (mean ± SE: 37.2% ± 7.4) and summer (34.2% ± 3.7) (Table 1). As for network structure, generality was significantly higher in summer (1.8 ± 0.1) than in spring (1.4 ± 0.08) (Table 1, Fig 1), but this effect disappeared after controlling for network size (Table 1). Interaction evenness showed a similar tendency (Table 1, Fig 1), which again disappeared after controlling for network size (Table 1). Vulnerability and H_2_’ showed no differences between seasons (Table 1, Fig 1).

## Discussion

The first objective of our study was to analyze seasonal changes in species richness, abundance and composition. The host community showed a strong seasonality. The spring community was almost exclusively composed of bees. Then, as the season progressed, wasps became increasingly speciose and abundant while bees maintained their species richness but became less abundant.

This pattern seems to be extensive to bee/wasp communities in general (not just cavity-nesters) and to other ecoregions besides the Mediterranean [38, 39, 58–61]. However, the strong seasonal changes in species composition in our host community were not only due to the addition of wasps late in the season. Temporal species turnover was also important within the bee guild. *Osmia* spp. were the first bees to appear in early spring, followed by other Osmiini (*Chelostoma* spp., *Hoplitis adunca*, *Heriades truncorum*) and *Hylaeus* spp., and, finally, the Megachilini (*Megachile* spp.), which occurred mostly in summer. As a result, the spring and summer host communities were drastically different.

The parasitoid community followed the dynamics of the host community. Abundance did not change seasonally, and species richness tended to increase from spring to summer, although this tendency failed significance for parasitoids. This result agrees with other studies showing a similar relationship between host and parasitoid community structure [10, 29, 52]. Seasonal changes in parasitoid composition also followed changes in host composition, as most parasitoid species showed some level of specificity at the host subgenus, genus or tribe level. Only 4 parasitoid species attacked both bees and wasps, whereas 10 attacked only bees and 5 only wasps.

Our second objective was to establish whether seasonal changes in taxonomic composition result in changes in community functional structure. Most of the traits considered showed a strong seasonal component, and these changes were not only due to the addition of wasp species in summer. The trends observed at the entire community level were maintained when only bees were considered. In addition, the increase in species richness in summer was accompanied by a clear increase in functional diversity, suggesting that species added in summer contribute a set of traits not present in spring (‘functional niche complementarity hypothesis’ [62]). Bee studies in tropical areas have similarly found changes in trait predominance between the dry and rainy seasons [18].

We found a seasonal decrease in body size for both the overall host and the bee communities. Body size has been shown to decrease from spring to summer in a regional bee fauna close to our study area [39]. This pattern was interpreted as a temporal extension of Bergmann’s rule, whereby species with larger body sizes are better equipped to generate and conserve body heat [63–65], and therefore can afford to be active in spring when temperatures are lower and floral resources are abundant [39]. The decrease in host body size had important consequences for the use of nesting resources, which showed a drastic decrease in diameter from spring to summer. Unlike bee and wasp species that excavate their nests, cavity-nesting species are totally dependent on pre-existing cavities, such as abandoned beetle burrows in dead wood, hollow stems, and abandoned bee/wasp nests [66]. Because preferred cavity diameter is correlated to body size, finding suitable cavities could be a limiting factor for populations of these species [67, 68].

In contrast to the host community, parasitoid functional traits did not vary seasonally. This could be explained in part by the longer mean activity periods of parasitoid species compared to host species (parasitoid: (mean±SE) 6.6±0.7 fortnights; bee hosts: 4.3±0.7; wasp hosts: 4.1±1.1). In fact, only 44% of the host species were found in the two seasons, compared to 63% of the parasitoid species. Nevertheless, in agreement with the bee/wasp dominance seasonal pattern, the parasitoid community showed a higher proportion of species attacking only bees in spring (69% ± 7%), and a higher proportion of species attacking both guilds or only wasps (70% ± 6% and 22% ± 5% respectively) in summer. Again, the seasonal increase in host species richness was accompanied by an increase in parasitoid functional diversity. Factors related to host biology, such as host habitat, food-plant type and feeding strategy are known to be important determinants of parasitoid community structure [69, 70] and, therefore, the structure of host communities is assumed to be an important driver of parasitoid functional diversity patterns. A study on island parasitoid assemblages along a worldwide latitudinal gradient found a positive relationship between island temperature and functional diversity [71]. The higher levels of energy available in warmer islands may have a positive effect on the availability of food and habitat resources [72], allowing the coexistence of a wider range of functions, and therefore greater functional diversity [71]. Even though host abundance in our study did not change between the colder (spring) and the warmer (summer) periods, we found a greater parasitoid functional diversity in summer, probably mediated by the greater host availability in this season.

The seasonal changes in functional structure found in our community involved functional traits potentially important for the establishment of host-parasitoid interactions (S1 Table). Consequently, we expected to find changes in parasitism rate and in host-parasitoid interaction networks (third objective of this study). However, none of these two expectations was fully met. Contrary to studies on other insect groups [73–75], parasitism rate in our community did not change seasonally. Other studies have found variation in parasitism rate at smaller temporal scales (weeks or months; [11, 55, 76]). Our community also shows important biweekly fluctuations in percent parasitism (S1 Table), but these changes show no seasonal pattern. Increased parasitism rate has been related to decreases in host abundance [73–75], and to increases in parasitoid species richness and abundance [11, 16, 55, 76]. The fact that host abundance and parasitoid abundance and richness in our study did not vary between seasons could explain the lack of seasonal changes in parasitism rate.

Regarding the relationship between community and network structure, some studies report no changes in network structure along temporal or spatial gradients despite important changes in community structure and/or composition [15, 16]. Our study shows seasonal changes in network structure (interaction evenness and generality increased from spring to summer), which are explained by the seasonal increase in network size (higher species richness in summer). The fact that only generality, but not vulnerability, showed a seasonal pattern may be consistent with the clear increase in host species richness in summer while parasitoid richness remained similar across seasons. The parallel increase of generality and taxonomic diversity seems to be a general pattern in antagonistic networks [30]. Other studies report parallel changes in community structure and network structure that, contrary to our results, remain significant even after controlling for network size or matrix size [9–14]. Interestingly, most of these studies found changes in both the richness and abundance of hosts and parasitoids, whereas our community showed no changes in host or parasitoid abundance.

Network structure could also be affected by seasonality through the effect of temperature on host-parasitoid interactions. Some studies have positively associated parasitoid activity and attack rates with temperature [77, 78], and parasitism rate, generality, vulnerability and interaction evenness have been found to be lower in colder environments [14, 16, 79]. However, the geographical gradients along which these studies were conducted are associated not only with temperature, but also with other abiotic and biotic factors, including structural and compositional changes in the host-parasitoid communities. Therefore, it is difficult to establish the role of temperature on interactions shifts along these gradients.

We found strong seasonal shifts in functional structure. However, these shifts did not appear to affect parasitism or network structure. Changes in network structure could be explained simply by changes in network size. These results are not in line with studies indicating that certain traits are related to species specialization [25–27], and consequently, to specialization at the network level. A possible explanation for the lack of relationship between changes in functional traits and changes in network structure in our study could be the existence of compensation between traits influencing network parameters in opposite directions. For example, the observed spring-to-summer decrease in host body size could lead to a decrease in vulnerability (as smaller hosts are not likely to be attacked by large parasitoids). However, the decrease in host body size was accompanied by a shift from mostly univoltine to mostly bivoltine hosts. Bivoltine hosts are active for longer periods and therefore are likely to be attacked by a greater range of parasitoid species, leading to increased vulnerability. Finally, although seasonal functional changes in our community seem not to have a clear reflection on network structure, the shift from a bee dominated community in spring to a wasp dominated community in summer is likely to have consequences on ecosystem function, with an emphasis on pollination function in spring and an emphasis in predation function in summer.

## Supporting information

S1 Table Functional trait descriptions. (PDF)

S2 Table Functional traits for each host species and literature source. (PDF)

S3 Table Functional traits for each parasitoid species and literature source. (PDF)

S4 Table Host and parasitoid species and their code numbers in Figs 1 and 2. (DOC)

S5 Table Variable transformations used in analyses. (DOC)

S6 Table General database. (XLS)

S1 Fig Biweekly fluctuations in percent parasitism. Mean percent parasitism for each fortnight (computing percent parasitism in each plot for each fortnight and then computing mean value considering all plots for each fortnight).
(TIF)
