# Supplementary material for: Seasonal dynamics in a cavity-nesting bee-wasp community: Shifts in composition, functional diversity and host-parasitoid network structure
Source: PLoS One. 2018 Oct 16;13(10):e0205854. doi: 10.1371/journal.pone.0205854 (PMC6191139; doi:10.1371/journal.pone.0205854)
Supplement: S1 Table — (PDF) [file pone.0205854.s001.pdf]

Osorio-Canadas, S., X. Arnan, E. Bassols, N. Vicens, and J. Bosch

S1 Table. Functional trait descriptions.

S1A Table. Hosts

Trait	Description	Type of variable	Potential importance for interactions with parasitoids	Source (see S2 Table)
Body size	Intertegular span (ITS), in mm. Highly correlated to body weight in bees [1, 2]. It has also been used for other insects [3].	Continuous	Host body size may constrain parasitoid size.	Own measures
Larval diet	Pollenivorous (bees) or carnivorous (wasps)	Categorical with two levels	Cleptoparasites may specialize on certain types of host provisions	Literature and own observations
Wintering stage	Adult or prepupa	Categorical with two levels	Species overwintering as adults are active earlier in the year than species wintering as prepupae (Bosch et al. 2001), thus conditioning the temporal overlap with parasitoid species.	Literature and own observations
Voltinism	Number of generations per year (univoltine or multivoltine)	Categorical with two levels	Multivoltine species usually have longer activity periods and therefore may be exposed to a greater range of parasitoids	Literature and own observations
Nest-building materials	Type of materials used by females use to build cell partitions and nest caps (mud, plant material, glandular secretions).	Categorical with three levels	Certain nesting materials (e.g., mud) may offer greater protection against parasitoids than others (e.g., leaves).	Own observations

1. Cane JH. Estimation of bee size using intertegular span (Apoidea). J. Kans. Entomol. Soc. 1987;60: 145–147.

2. Peters MK, Peisker J, Steffan-Dewenter I, Hoiss B. Morphological traits are linked to the cold performance and distribution of bees along elevational gradients. J. Biogeogr. 2016;43: 2040-2049. doi:10.1111/jbi.12768.

3. Chifflet R, Klein EK, Lavigne C, Le Féon L, Ricroch AE, Lecomte J, et al. Spatial scale of insect-mediated pollen dispersal in oilseed rape in an open agricultural landscape. Journal of Applied Ecology. 2011;48:689–696. doi:10.1111/j.1365-2664.2010.01904.x.

S1B Table. Parasitoids

**Trait**	**Description**	**Type of variable**	**Potential importance for interactions with parasitoids**	**Source** (see S2 Table)
Body size	Measured as body length, in mm	Continuous	Parasitoid size may constrain suitable host size.	Literature and own measures
Parasitic behavior	parasitoids, cleptoparasites, and scavenger/predators	Categorical with three levels	Cleptoparasites are expected to have a narrower diet breadth than parasitoids and, especially, scavengers	Literature
Wintering stage	Adult or immature	Categorical with two levels	Species overwintering as adults tend to be active earlier in the year than species wintering immatures, thus conditioning the temporal overlap with host species.	Literature
Voltinism	Number of generations per year (univoltine or multivoltine)	Categorical with two levels	Multivoltine species usually have longer activity periods and therefore may have access to a greater range of hosts	Literature
Gregariousness	Solitary (only one parasitoid individual develops per host individual) or gregarious (several parasitoids develop)	Categorical with two levels	In multivoltine species, the capacity to increase percent parasitism is likely to be greater in gregarious species	Literature and own observations
