# Supplementary material for: Seasonal dynamics in a cavity-nesting bee-wasp community: Shifts in composition, functional diversity and host-parasitoid network structure
Source: PLoS One. 2018 Oct 16;13(10):e0205854. doi: 10.1371/journal.pone.0205854 (PMC6191139; doi:10.1371/journal.pone.0205854)
Supplement: S2 Table — (PDF) [file pone.0205854.s002.pdf]

S2 Table. Functional traits for each Host species and literature source.

Hosts species	**Family**	**Larval diet**	**Body size (ITS,mm)**	**Wintering stage**	**Voltinism**	**Nest-building material**
Hylaeus communis	COLLETIDAE	POLLENIVOROUS	1,27	PREPUPA [1]	BIVOLTINE [14-16]	SECRETIONS [2,26]
Hylaeus signatus	COLLETIDAE	POLLENIVOROUS	1,66	PREPUPA [1-2]	BIVOLTINE [14-15]	SECRETIONS [2,26]
Hylaeus taeniolatus	COLLETIDAE	POLLENIVOROUS	1,05	PREPUPA [1-2]	BIVOLTINE [14]	SECRETIONS [2,26]
Chelostoma campanularum	MEGACHILIDAE	POLLENIVOROUS	0,91	PREPUPA [3-4]	UNIVOLTINE [14,9]	MUD
Chelostoma emarginata	MEGACHILIDAE	POLLENIVOROUS	1,48	PREPUPA [3-4]	UNIVOLTINE [14]	MUD
Chelostoma florisomne	MEGACHILIDAE	POLLENIVOROUS	1,67	ADULT [1,5-6]	UNIVOLTINE [14,9]	MUD
Heriades truncorum	MEGACHILIDAE	POLLENIVOROUS	1,37	PREPUPA [1,5]	UNIVOLTINE [14,16]	PLANT MATERIAL
Hoplitis adunca	MEGACHILIDAE	POLLENIVOROUS	2,48	PREPUPA [3]	UNIVOLTINE [3][a]	MUD
Megachile apicalis	MEGACHILIDAE	POLLENIVOROUS	2,29	PREPUPA [7]	UNIVOLTINE [14]	PLANT MATERIAL
Megachile centuncularis	MEGACHILIDAE	POLLENIVOROUS	2,71	PREPUPA [1]	BIVOLTINE [14-16]	PLANT MATERIAL
Megachile rotundata	MEGACHILIDAE	POLLENIVOROUS	2,45	PREPUPA [8]	UNIVOLTINE [14]	PLANT MATERIAL
Osmia bicornis (=rufa)	MEGACHILIDAE	POLLENIVOROUS	3,08	ADULT [3]	UNIVOLTINE [2-3,14]	MUD
Osmia caerulescens	MEGACHILIDAE	POLLENIVOROUS	2,17	ADULT [9]	BIVOLTINE [9,14,16]	PLANT MATERIAL
Osmia cornuta	MEGACHILIDAE	POLLENIVOROUS	4,12	ADULT [3]	UNIVOLTINE [2-3,14]	MUD
Osmia fulviventris	MEGACHILIDAE	POLLENIVOROUS	2,92	ADULT [3]	UNIVOLTINE [2-3,14]	PLANT MATERIAL
Osmia submicans	MEGACHILIDAE	POLLENIVOROUS	2,07	ADULT [3]	UNIVOLTINE [2-3,14]	PLANT MATERIAL
Passaloecus spp (mostly *corniger*, some *eremita* & *gracilis*)	CRABRONIDAE	CARNIVOROUS	0,87	PREPUPA [2]	BIVOLTINE [2,9,17]	PLANT MATERIAL
Pison atrum	CRABRONIDAE	CARNIVOROUS	1,78	PREPUPA [2,10-12]	BIVOLTINE [18-19]	MUD
Psenulus fuscipennis	CRABRONIDAE	CARNIVOROUS	1,49	PREPUPA [2,10-12]	BIVOLTINE [9,20-21]	SECRETIONS [27]
Solieriella compedita	CRABRONIDAE	CARNIVOROUS	0,87	PREPUPA [2]	BIVOLTINE [2]	PLANT MATERIAL
Trypoxylon figulus	CRABRONIDAE	CARNIVOROUS	1,49	PREPUPA [2]	BIVOLTINE [2,9,22]	MUD
Trypoxylon spp (mostly *clavicerum*, some *minus*)	CRABRONIDAE	CARNIVOROUS	0,76	PREPUPA [2]	BIVOLTINE [2,9,23]	MUD
Alastor atropos	VESPIDAE (EUMENINAE)	CARNIVOROUS	1,31	PREPUPA [2,10-12]	BIVOLTINE [2][b]	MUD [28]
Euodynerus posticus	VESPIDAE (EUMENINAE)	CARNIVOROUS	2,22	PREPUPA [2]	BIVOLTINE [2,24][b]	MUD
Microdynerus nugdunensis	VESPIDAE (EUMENINAE)	CARNIVOROUS	1,19	PREPUPA [2,10-12]	BIVOLTINE [13][b]	MUD
Microdynerus timidus	VESPIDAE (EUMENINAE)	CARNIVOROUS	0,90	PREPUPA [2,10-12]	BIVOLTINE [13][b]	MUD
Isodontia mexicana	SPHECIDAE	CARNIVOROUS	2,20	PREPUPA [2]	BIVOLTINE [2,25]	PLANT MATERIAL

REFERENCES

1. Fründ J, Zieger SL, Tscharntke T. Response diversity of wild bees to overwintering temperatures. Oecologia. 2013;173: 1639–1648. DOI 10.1007/s00442-013-2729-1
2. Krombein KV. Trap-nesting wasps and bees: life histories, nests, and associates. Washington: Smithsonian Press; 1967.
3. Bosch J, Maeta Y, Rust R. A Phylogenetic Analysis of Nesting Behavior in the Genus Osmia (Hymenoptera: Megachilidae). Ann. Entomol. Soc. Am. 2001;94(4): 617-627.
4. Westrich P. Die Wildbienen Baden-Württembergs. Stuttgart: Ulmer Verlag; 1989.
5. Haider M. Evolution of host range in pollen generalist bees: insights from the subgenus Osmia (Megachilidae: Osmiini). PhD Thesis. Universität Konstanz. 2013. Availble from: doi:10.3929/ethz-a-009752593
6. Rozen JG, Praz, CJ. Mature Larvae and Nesting Biologies of Bees Currently Assigned to the Osmiini (Apoidea: Megachilidae). AMERICAN MUSEUM NOVITATES 2016;3864. Available from: http://hdl.handle.net/2246/6669.
7. Bosch J, Vicens N, Blas M. Análisis de 10s nidos de algunos Megachilidae nidificantes en cavidades preestablecidas (Hymenoptera, Apoidea). Orsis. 1993;8: 53-63.
8. Kemp WP, Bosch J, Dennis B. Oxygen Consumption During the Life Cycles of the Prepupa Wintering Bee Megachile rotundata and the Adult-Wintering Bee Osmia lignaria (Hymenoptera: Megachilidae). Ann. Entomol. Soc. Am. 2004,97(1): 161-170. doi.10.1603/0013-8746(2004)097[0161:OCDTLC]2.0.CO;2
9. Bees, Wasps & Ants Recording Society (BWARS). 2017. [cited 17 March 2017]. Available from: http://www.bwars.com.
10. Guido G. Studi di un entomologo sugli imenotteri superiori. Bologna: Calderini; 1961.
11. O'Neill K. Solitary Wasps. Behavior and Natural History. Ithaca: Comstock Publishing Associates. Cornell Series in Arthropod Biology; 2001.
12. Hunt H. The Evolution of Social Wasps. Oxford: Oxford University Press; 2007. doi:10.1093/acprof:oso/9780195307979.001.0001
13. Arens W. Die solitären Faltenwespen der Peloponnes (Hymenoptera: Vespidae: Raphiglossinae, Eumeninae) – 2 Teil. Linzer biologische Beiträge. 2012;44(2): 933-971.
14. Gogala A. Bee fauna of Slovenia. 2017. [cited 17 March 2017]. Available from: http://www2.pms-lj.si/andrej/apoidea.htm.
15. Martin, H-J. Wildbienen (Biologie, Arten, Schutz). [cited 17 March 2017]. 2017. Available from: http://wildbienen.de
16. Diekötter T, Peter F, Jauker B, Wolter, V, Jauker F. Mass-flowering crops increase richness of cavity-nesting bees and wasps in modern agro-ecosystems. GCB Bioenergy. 2014;6: 219-226. doi:10.1111/gcbb.12080.
17. Grandi, G. Contributi alla conoscenza degli Imenottori melliferi e predatori, XVI. Boll. 1st. Ent. Univ. Bologna. 1937;9: 253-346.
18. Gess SK, Gess FW. Wasps and bees of southern africa. Sanbi biodiversity series 24. Pretoria: SANBI Publishing; 2014.
19. Harris AC. Sphecidae (Insecta: Hymenoptera). Fauna of New Zealand 32. Lincoln (Canterbury, NZ): Manaaki Whenua Press; 1994.
20. Bitch J, DOLLFUSS H, BOUCEK Z, SCHMIDT K, SCHMID-EGGER C, Fernández-GAYUBO S, et al. Fauna de France 86. Hyménoptères Sphecidae d'Europe Occidentale. Volume3. Première édition. Paris: Fédération Française des Sociétés de Sciences Naturelles; 2001.
21. Bohart RM, Menke AS. Sphecid Wasps of the World: A Generic Revision. Berkeley : University of California Press; 1976.
22. Jacob-Remacle A. Abeilles et guêpes de nos jardins. Gembloux: Duculot. 1989.Publications de la Société Linnéenne de Lyon.
23. Dorow WHO. Die Hautflügler (Hymenoptera) des Naturwaldreservats Goldbachs- und Ziebachsrück (Hessen). Untersuchungszeitraum 1994–1996. In: Dorow WHO, Blick T, Kopelke J-P: Naturwaldreservate in Hessen, Band 11/2.2 Goldbachs- und Ziebachsrück. Zoologische Untersuchungen 1994–1996, Teil 2. Mitteilungen der Hessischen Landesforstverwaltung. 2010; 46: 111-217.
24. Eaton ER, Kaufman K. Kaufman field guide to insects of North America. Boston: Houghton Mifflin Harcourt; 2007.
25. Fateryga AV, Protsenko YV, Zhidkov VY. Isodontia mexicana (Hymenoptera, Sphecidae), a new invasive wasps species in the fauna of Ukraine reared from trap-nests in the Crimea. Vestnik zoologii. 2014;48(2): 185–188. doi: 10.2478/vzoo-2014-0020
26. Stephen W, Bohart GE, Torchio PF. The Biology and External Morphology of Bees. Corvallis (OR): Oregon State University; 1969.
27. Malyshev Sl. Genesis of the Hymenoptera and phases of their evolution. London: Methuen; 1966.
28. EBRAHIMI E, CARPENTER JM. Catalog of the vespid wasps of Iran. Zootaxa. 2008;1785: 1–42.

---

[a] Parsivoltine [3] but considered UNIVOLTINE to our analyses

[b] Considered BIVOLTINE also following L.Castro (per. comm.)
