# Supplementary material for: Seasonal dynamics in a cavity-nesting bee-wasp community: Shifts in composition, functional diversity and host-parasitoid network structure
Source: PLoS One. 2018 Oct 16;13(10):e0205854. doi: 10.1371/journal.pone.0205854 (PMC6191139; doi:10.1371/journal.pone.0205854)
Supplement: S3 Table — (PDF) [file pone.0205854.s003.pdf]

S3 Table. Functional traits for each Parasitoid species and literature source.

Parasitoid species	**Order / Infraclass**	**Parasitic behaviour**	**Body size length (mm)**	**Wintering stage**	**Voltinism**	**Gregariousness**
Chaetodactylus osmiae	ACARI	CLEPTOPARASITE [1]	0,6 [18]	INMATURE [4]	MULTIVOLTINE [4,15]	GREGARIOUS [4]
Pyemotes ventricosus	ACARI	PARASITOID [2]	1,1 [19]	ADULT [25]	MULTIVOLTINE [25]	GREGARIOUS [2]
Ptinus pyrenaeus	COLEOPTERA	PRED/SCAVENGER [3-4]	5,5 [3]	ADULT [3-4]	UNIVOLTINEV [4]	SOLITARY [3]
Ptinus sexpunctatus	COLEOPTERA	PRED/SCAVENGER [4]	3,5 [7]	ADULT [3-4]	UNIVOLTINEV [4]	SOLITARY [16]
Trichodes alvearius	COLEOPTERA	PRED/SCAVENGER [5]	13,5 [4,20]	INMATURE [4-5]	UNIVOLTINEV [5]	SOLITARY [5,16]
Anthrax anthrax	DIPTERA	PARASITOID [4]	10 [8]	INMATURE [2,26]	UNIVOLTINEV [4]	SOLITARY [4,16]
Cacoxenus indagator	DIPTERA	CLEPTOPARASITE [1]	3,3 [4]	INMATURE [4]	UNIVOLTINEV [4]	GREGARIOUS [4]
Sarcophagidae sp.1	DIPTERA	CLEPTOPARASITE [6-8]	5,3	INMATURE [26-28]	UNIVOLTINEV [8,27]	GREGARIOUS [34]
Sarcophagidae sp.2	DIPTERA	CLEPTOPARASITE [6-8]	7,8	INMATURE [26-28]	UNIVOLTINEV [8,27]	GREGARIOUS [34]
Chrysis ignita	HYMENOPTERA	CLEPTOPARASITE [4,9]	7,5 [8,21]	INMATURE [9,26]	MULTIVOLTINE [9]	SOLITARY [9]
Gasteruption sp	HYMENOPTERA	CLEPTOPARASITE [8,10-11]	13 [29]	INMATURE [29]	UNIVOLTINEV [8]	SOLITARY [11]
Melittobia acasta	HYMENOPTERA	PARASITOID [4,12-13]	1,25 [7-8,15]	INMATURE [4,26]	MULTIVOLTINE [32]	GREGARIOUS [4,15-16]
Monodontomerus obsoletus	HYMENOPTERA	PARASITOID [4]	4,35 [8]	INMATURE [4]	MULTIVOLTINE [4]	GREGARIOUS [4,15-16]
Omalus (=Pseudomalus) auratus	HYMENOPTERA	PARASITOID [9]	4,5 [21]	INMATURE [9,26]	MULTIVOLTINE [9]	SOLITARY [9]
Hybomischos (=Perithous) septemcinctorius	HYMENOPTERA	PARASITOID [4]	7,5 [18]	INAMATURE [30]	MULTIVOLTINE [30,33]	SOLITARY [35]
Sapyga 5-punctata	HYMENOPTERA	CLEPTOPARASITE [14-16]	10,5 [22]	ADULT [15]	UNIVOLTINEV [8,14-15]	SOLITARY [15]
Stelis breviuscula	HYMENOPTERA	CLEPTOPARASITE [16]	5,5 [23]	INMATURE [15]	UNIVOLTINEV [8,14-15]	SOLITARY [15-16]
Trichrysis cyanea	HYMENOPTERA	CLEPTOPARASITE [17]	6 [21]	INMATURE [9,26]	MULTIVOLTINE [9]	SOLITARY [17]
Plodia interpunctella	LEPIDOPTERA	PRED/SCAVENGER [4]	9 [24]	INMATURE [31]	MULTIVOLTINE [7,31]	GREGARIOUS [36]

REFERENCES

1. Vicens N, Bosch J, Blas M. Analisis de los nidos de algunas Osmias nidificantes en cavidades preestablecidas. Orsis. 1993;8: 41-52.
2. Krombein KV. Trap-nesting wasps and bees: life histories, nests, and associates. Washington: Smithsonian Press; 1967.
3. Bellés X, Bosch J. Nuevos datos taxonómicos, corológicos y biológicos sobre Ptinus (Gynopterus) pyrenaeus Pic. (Coleoptera, Ptinidae). Orsis. 1994;9: 77-84.
4. Krunić M, Stanisavljević L, Pinzauti M, Felicioli A. The accompanying fauna of Osmia cornuta and Osmia rufa and effective measures of protection. Bull. Insectol. 2005;58(2): 141-152.
5. Carré S. Biologie de deux prédateurs de l'abeille solitaire Megachile rotundata F. (Hymenoptera, Megachilidae): Trichodes alvearius F. et Trichodes apiarius L. (Coleoptera, Cleridae).- Apidologie. 1980;11 (3): 255-295.
6. Stephen W, Bohart GE, Torchio PF. The Biology and External Morphology of Bees. Corvallis (OR): Oregon State University; 1969.
7. Identification, Images, & Information For Insects, Spiders & Their Kin For the United States & Canada (Bugguide).2017. [cited 17 March 2017]. Iowa State University. Department of Entomology.Available from: http://bugguide.net/
8. Martin, H-J. Wildbienen (Biologie, Arten, Schutz). [cited 17 March 2017]. 2017. Available from: http://wildbienen.de
9. Wiśniowski P. Cuckoo-wasps (Hymenoptera: Chrysididae) of Poland. Diversity, identification, distribution. Ojców: Ojcow National Park: 2015.
10. Gauld ID. The evaniomorph parasitoid families. Gasteruptiidae, p. 193–195. In: Hanson PE, Gauld ID (eds). The Hymenoptera of Costa Rica. Oxford: Oxford University Press; 1995.
11. Funk W. Insekten box. 2017. [cited 20 March 2017]. Available from: http://www.insektenbox.de/
12. Krunic MD, Brajkovic MM, Mihajlovic LS. Management and utilization of Osmia comuta Latr for orchard pollination in Yugoslavia. Sixth Int Symp on Pollination. Acta Hortic. 1991;288: 190-193.
13. de Wael L, de Greef M, van Laere O. Biology and Control of Melittobia Acasta. Bee World. 1195;76(2): 72-76. doi: 10.1080/0005772X.1995.11099244.
14. Bees, Wasps & Ants Recording Society (BWARS). 2017. [cited 17 March 2017]. Available from: http://www.bwars.com.
15. Bosch J, Kemp WP. How to Manage the Blue Orchard Bee As an Orchard Pollinator. Beltsville: National Agricultural Library; 2001.
16. Westrich P. Die Wildbienen Baden-Württembergs. Stuttgart: Ulmer Verlag; 1989.
17. Rosa P. I Crisidi (Hymenoptera, Chrysididae) della Valle d'Aosta. Check-list e note introduttive. Revue Valdôtaine d'Histoire Naturelle. 2002; 56: 63-70.
18. Galerie du Monde des insectes. 2017. [cited 20 March 2017]. Available from: https://www.galerie-insecte.org/
19. PESTIUM.UK. Europe's largest scientific bug site. 2017. [cited 20 March 2017]. Available from: http://www.pestium.uk/bedbugs-bites-stings-and-itches/small-mites/water-mites/
20. Käfer Europas. 2017. [cited 20 March 2017]. Available from: http://www.coleo-net.de/coleo/texte/trichodes.htm
21. Mingo E. Fauna Iberica. Vol. 6: Hymenoptera, Chrysididae. Madrid: Consejo Superior de Investigaciones Científicas; 1994.
22. Richards, OW. Handbooks for the Identification of British Insects. Hymenoptera, Aculeata. Vespoidea, Scolioidea and Sphecoidea. Vol 6. Part 3(b). London: Royal Entomological Society of London; 1980.
23. Rasmont, P. et al. Atlas Hymenoptera. 2017. [cited 21 March 2017]. Available from: http://www.atlashymenoptera.net/
24. Lyon WF. 2006. Ohio State University Insect and Pest Fact Sheet. HYG-2089-97: Indianmeal Moth. [31 August 2017]. Available from: https://www.maine.gov/dacf/php/gotpests/bugs/documents/indian-meal-moth-ohio.pdf
25. Vega F, Kaya H. Insect Pathology. 2nd ed. London: Academic Press; 2012.
26. Univeristy of Toronto. 2017. [cited 21 March 2017]. Available from: https://tspace.library.utoronto.ca/bitstream/1807/30672/1/Darling_Hallett2007NESTS.pdf
27. Clausen CP. Entomophagous Insects. NY. & London: Hafner Publ. Co.; 1962 (reimpr.).
28. van Emden FI. Handbooks for the Identification of British Insects. Vol.X. Part 4-(a). Diptera Cyclorrhapha Calyptrata (I). Section a. Calliphoridae and Tachinidae. London: Royal Entomological Society of London; 1954.
29. van Achterberg C, Talebi AA. Review of Gasteruption Latreille (Hymenoptera, Gasteruptiidae) from Iran and Turkey, with the description of 15 new species. ZooKeys. 2014;458: 1–187. doi: 10.3897/zookeys.458.8531.
30. Morley C. Ichneumonologia Britannica, iii. The Ichneumons of Great Britain. Pimplinae. London: H. & W. Brown; 1953.
31. Road K. 1991. Ohio State University Extension Fact Sheet. Entomology. Columbus, OH 43210-1000. Available from: https://www.maine.gov/dacf/php/gotpests/bugs/documents/indian-meal-moth-ohio.pdf
32. Hobbs GA, Krunic MD. Comparative behavior of three chalcidoid (Hymenoptera) parasites of the alfalfa leafcutter bee, Megachile rotundata, in the laboratory. Canadian Entomologist. 1971;103(5): 674-685.
33. Fitton MG, Shaw MR, Gauld ID. Handbooks for the Identification of British Insects. Pimpline Icneumon-flies Hymenoptera. Vol. 7, Part 1. London: Royal Entomological Society of London; 1988.
34. Pape T. The Sarcophagidae (Diptera) of Fennoscandia and Denmark. Fauna entomologica Scandinavica, 19. Leiden/Copenhagen: E.J. Brill/Scandinavian Science Press Ltd; 1987.
35. Tormos J, Asís JD, Elfa JS. Description of the final larva of Perithous scurra, with comments on its morphological characters. (HYMENOPTERA: ICHNEUMONIDAE, PIMPLINAE). Florida Entomol. 1999;82(2): 333-339.
36. Sambaraju KR, Donelson SL, Bozic J, Phillips TW. Oviposition by Female Plodia interpunctella (Lepidoptera: Pyralidae): Description and Time Budget Analysis of Behaviors in Laboratory Studies. Insects. 2016;7, 4. doi:10.3390/insects7010004.
