# Supplementary material for: Seasonal dynamics in a cavity-nesting bee-wasp community: Shifts in composition, functional diversity and host-parasitoid network structure
Source: PLoS One. 2018 Oct 16;13(10):e0205854. doi: 10.1371/journal.pone.0205854 (PMC6191139; doi:10.1371/journal.pone.0205854)
Supplement: S4 Table — (DOC) [file pone.0205854.s004.doc]

| **S4 Table. Host and Parasitoid species and their code numbers in Figs 1 and 2.** | | | |
| --- | --- | --- | --- |
| **Code in**  **Figs 1 and 2** | **Family** | | ***Species*** |
| **BEE HOSTS** | | | |
| 1 | COLLETIDAE | | *Hylaeus communis* |
| 2 | COLLETIDAE | | *Hylaeus signatus* |
| 3 | COLLETIDAE | | *Hylaeus taeniolatus* |
| 4 | MEGACHILIDAE | | *Chelostoma campanularum* |
| 5 | MEGACHILIDAE | | *Chelostoma emarginata* |
| 6 | MEGACHILIDAE | | *Chelostoma florisomne* |
| 7 | MEGACHILIDAE | | *Heriades truncorum* |
| 8 | MEGACHILIDAE | | *Hoplitis adunca* |
| 9 | MEGACHILIDAE | | *Megachile apicalis* |
| 10 | MEGACHILIDAE | | *Megachile centuncularis* |
| 11 | MEGACHILIDAE | | *Megachile rotundata* |
| 12 | MEGACHILIDAE | | *Osmia bicornis* |
| 13 | MEGACHILIDAE | | *Osmia caerulescens* |
| 14 | MEGACHILIDAE | | *Osmia cornuta* |
| 15 | MEGACHILIDAE | | *Osmia fulviventris* |
| 16 | MEGACHILIDAE | | *Osmia submicans* |
| **WASP HOSTS** | | | |
| 17 | CRABRONIDAE | *Passaloecus* spp*.*^1^ | |
| 18 | CRABRONIDAE | *Pison atrum* | |
| 19 | CRABRONIDAE | *Psenulus fuscipennis* | |
| 20 | CRABRONIDAE | *Solierella compedita* | |
| 21 | CRABRONIDAE | *Trypoxylon figulus* | |
| 22 | CRABRONIDAE | *Trypoxylon* spp*.*^2^ | |
| 23 | VESPIDAE | *Alastor atropos* | |
| 24 | VESPIDAE | *Euodynerus posticus* | |
| 25 | VESPIDAE | *Microdynerus nugdunensis* | |
| 26 | VESPIDAE | *Microdynerus timidus* | |
| 27 | SPHECIDAE | *Isodontia mexicana* | |
| **PARASITOIDS** | | | |

| **Code in**  **Figs 1 and 2** | **Order/Infraclass** | ***Parasitoid species*** |
| --- | --- | --- |
| 28 | ACARI | *Chaetodactylus osmiae* |
| 29 | ACARI | *Pyemotes ventricosus* |
| 30 | COLEOPTERA | *Ptinus pyrenaeus* |
| 31 | COLEOPTERA | *Ptinus sexpunctatus* |
| 32 | COLEOPTERA | *Trichodes alvearius* |
| 33 | DIPTERA | *Anthrax anthrax* |
| 34 | DIPTERA | *Cacoxenus indagator* |
| 35 | DIPTERA | Sarcophagidae sp.1 |
| 36 | DIPTERA | Sarcophagidae sp.2 |
| 37 | HYMENOPTERA | *Chrysis ignita* |
| 38 | HYMENOPTERA | *Gasteruption* sp. |
| 39 | HYMENOPTERA | *Melittobia acasta* |
| 40 | HYMENOPTERA | *Monodontomerus obsoletus* |
| 41 | HYMENOPTERA | *Omalus auratus* |
| 42 | HYMENOPTERA | *Perithous septemcinctorius* |
| 43 | HYMENOPTERA | *Sapyga quinquepunctata* |
| 44 | HYMENOPTERA | *Stelis breviuscula* |
| 45 | HYMENOPTERA | *Trichrysis cyanea* |
| 46 | LEPIDOPTERA | *Plodia interpunctella* |

^1^ Mostly *Passaloecus corniger* along with some *P. eremita* and *P. gracilis*.

^2^ Mostly *Trypoxylon clavicerum* along with some *T. minus*.
