# Supplementary material for: Seasonal dynamics in a cavity-nesting bee-wasp community: Shifts in composition, functional diversity and host-parasitoid network structure
Source: PLoS One. 2018 Oct 16;13(10):e0205854. doi: 10.1371/journal.pone.0205854 (PMC6191139; doi:10.1371/journal.pone.0205854)
Supplement: S5 Table — (DOC) [file pone.0205854.s005.doc]

| **S5 Table. Variable transformations used in analyses.** | | |
| --- | --- | --- |
| **Transformation** | **Variable** | **Table**  **where transformed variables are included in the main text** |
| square root | Host abundance | 1 |
|  | Parasitoid abundance | 1 |
|  | Trait average of Percent plant material as nest-building material (Hosts) | 3A |
| log10 | Percent parasitism | 1 |
|  | Parasitoid abundance controlled by Host richness | 1 |
|  | Percent parasitism controlled by Host richness | 1 |
|  | Generality controlled by Networksize | 1 |
|  | Functional dispersion of Body size (all Hosts and only bees) | 3A and 3B |
|  | Functional dispersion of Percent prepupa (wintering stage) (only bees) | 3B |
| second power | Functional dispersion of Voltinism (Parasitoids) | 3C |
| third power | Functional dispersion of Parasitic behavior (Parasitoids) | 3C |
| arcsine(squared root) | Trait average of Percent mud as nest-building material (Hosts) | 3A |
