# Supplementary figures and images for: Seasonal dynamics in a cavity-nesting bee-wasp community: Shifts in composition, functional diversity and host-parasitoid network structure
Source: PLoS One. 2018 Oct 16;13(10):e0205854. doi: 10.1371/journal.pone.0205854 (PMC6191139; doi:10.1371/journal.pone.0205854)

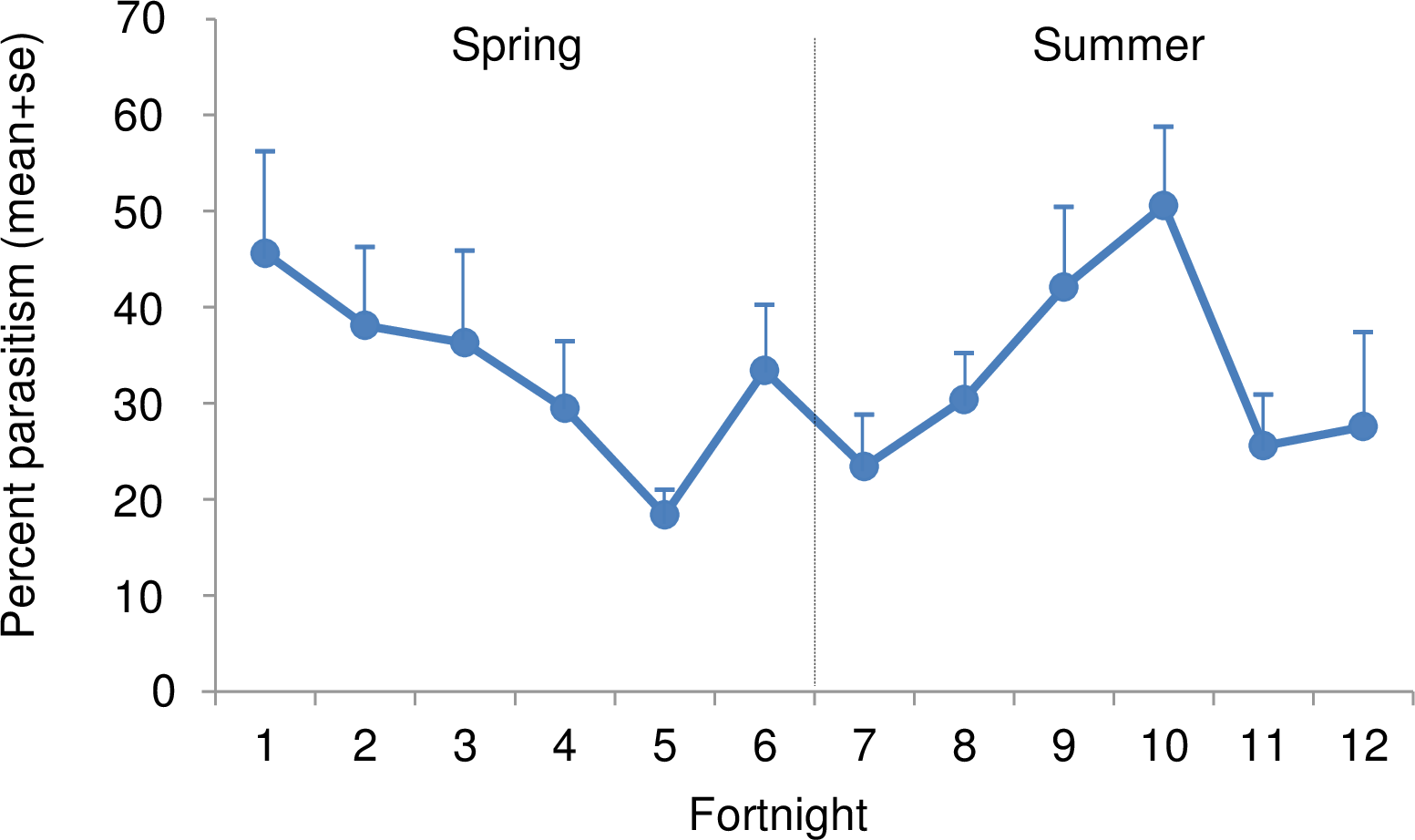

Supplement: S1 Fig — Mean percent parasitism for each fortnight (computing percent parasitism in each plot for each fortnight and then computing mean value considering all plots for each fortnight). (TIF) [file pone.0205854.s007.tif]
